# Reducing Environmental Tobacco Smoke Exposure of Preschool Children: A Randomized Controlled Trial of Class-Based Health Education and Smoking Cessation Counseling for Caregivers

**DOI:** 10.3390/ijerph120100692

**Published:** 2015-01-13

**Authors:** Yun Wang, Zhiqiang Huang, Mei Yang, Fuzhi Wang, Shuiyuan Xiao

**Affiliations:** 1 Department of Social Medicine and Health Management, School of Public Health, Central South University, Changsha 410078, Hunan, China; E-Mails: xjwangyun@csu.edu.cn (Y.W.); ym8342@163.com (M.Y.); bengbu_wangfuzhi@126.com (F.W.); 2 School of Nursing, Xinjiang Medical University, Urumqi 830000, Xinjiang, China; 3 Academy of Inspection and Quarantine, Changsha 410004, Hunan, China; E-Mail: huangzqq@126.com

**Keywords:** environmental tobacco smoke (ETS), young children, intervention, urinary cotinine

## Abstract

*Objectives*: To assess counseling to caregivers and classroom health education interventions to reduce environmental tobacco smoke exposure of children aged 5–6 years in China. *Methods*: In a randomized controlled trial in two preschools in Changsha, China, 65 children aged 5–6 years old and their smoker caregivers (65) were randomly assigned to intervention (*n* = 33) and control (no intervention) groups (*n* = 32). In the intervention group, caregivers received self-help materials and smoking cessation counseling from a trained counselor, while their children were given classroom-based participatory health education. Children’s urinary cotinine level and the point prevalence of caregiver quitting were measured at baseline and after 6 months. *Results*: At the 6-month follow-up, children’s urinary cotinine was significantly lower (*Z* = –3.136; *p* = 0.002) and caregivers’ 7-day quit rate was significantly higher (34.4% *versus* 0%) (*p* < 0.001; adjusted OR = 1.13; 95% CI: 1.02–1.26) in the intervention than control group. *Conclusions*: Helping caregivers quitting smoke combined with classroom-based health education was effective in reducing children’s environmental tobacco smoke exposure. Larger-scale trials are warranted.

## 1. Introduction 

Environmental tobacco smoke (ETS) is one of the most common indoor air pollutants. ETS is composed of two components: secondhand smoke (SHS) and thirdhand smoke (THS) [1,2]. Globally, 40% of children are regularly exposed to SHS, and 28% of deaths that result from ETS occur in children [3]. Recently, research has shown that the link between passive smoking and disease is mediated by many chemicals in ETS, including carcinogens such as benzene, 1,3-butadiene, benzo[a]pyrene and 4-(methylnitrosamino)-1-(3-pyridyl)-1-butanone, amongst others. Some research has been conducted to assess the links between benzene exposure in children, ETS and the risk of lymphohematopoietic disorders [4,5,6]. Children exposed to ETS are at an increased risk for sudden infant death syndrome, acute respiratory infections, ear problems, and more severe asthma [7,8,9,10,11]. China is the world’s largest producer and consumer of tobacco, with over 350 million smokers [12]. A survey from the Inner Mongolia Autonomous Region found that more than half of children aged 4–7 years lived with at least one smoker and more than one-third of smokers smoked in front of them [13]. 

Strategies to reduce children’s exposure to tobacco smoke fall into two categories: helping caregivers to quit smoke, and keeping children away from ETS. Interventions to quit smoking include self-help materials, face-to-face counseling, telephone counseling, cessation medications, and feedback on urinary cotinine levels. The self-help materials included a guide with tips and action-oriented strategies. 

Most participants, however, did not quit smoking and therefore did not protect their children from ETS exposure. Interventions to help caregivers smoke away from their children may yield important health benefits. For example, one study found a statistically significant effect of reducing ETS exposure by mothers at home after counseling compared to families that did not receive counseling [14]. The rate of exposure of children may be significantly higher in China compared to other countries. Yao *et al*. found in 2008 that the average at-home ETS exposure rate for children aged 0–18 years old was 68% among six rural counties in China [15]. 

As young children are dependent on their caregivers for protection from ETS, interventions designed to reduce ETS exposure in this age group must emphasize education for caregivers. Given the importance of preventing pediatric ETS exposure early, it is important to focus on reducing the exposure of young children to ETS, both from the viewpoint of the individual child, their family and society as a whole from reduced future healthcare and absenteesism costs [16]. However, ETS interventions for children are very rare, especially for preschoolers. A study in Iran randomly assigned ETS-exposed children aged 8–12 years old to a parent intervention group and a children’s intervention group. Their findings showed that educating the children was more effective than training the parents to decrease ETS exposure [17]. However, other intervention studies have been conducted in preschoolers, such as sun protection [16], and school-based participatory health education programs have been conducted for malaria control [18], obesity intervention [19] and environmental education [20]. Some research into children health education has been developed according to social cognitive theory [16,21], other studies were based on cognitive behavioral therapy concepts [22], while others did not use a specific psychological theory as the basis of the study [19,20].

Older children may also be educated to withdraw voluntarily from ETS exposure in spaces where they live, play, and learn. It may be helpful if children are not merely recipients of health education, but also contribute to ETS intervention by playing the role of health change agents in the family. The approach of engaging children aged 5–6 years old as health messengers was not employed in the current study in order to confirm the effectiveness of the strategy, with the consideration that sociocultural factors in the different areas might influence its effectiveness. 

In this study we aimed to evaluate the effectiveness of helping caregivers to quit smoking and of providing participatory health education for children in reducing ETS exposure of aged children 5–6 years old in Changsha city, China. We hypothesized that smoking cessation counseling for caregivers and classroom-based education for children can produce behavior change in caregivers, leading to a reduction in children’s exposure to ETS, and a decrease the urinary cotinine level of the children. 

## 2. Methods

### 2.1. Participants and Study Design

The study was a randomized controlled intervention trial. The study participants were selected using two-stage, simple random sampling. During the first stage, six districts and one county in Changsha were identified; one district was then drawn at random. In the second stage, preschools registered in Changsha were identified in a single district, from which two preschools were selected at random. Participants were recruited through the Changsha Center for Disease Control and Prevention between December 2012 and January 2013. The entire intervention procedure took place from January to June, 2013.

All children from the selected preschools and their caregivers received information about the aims and plans of our study. During the process of recruitment, when >1 parent/caregiver in a family smoked, the primary caregiver was selected for participation in the study. The primary caregiver was designated as the person who was a smoker and mainly cared for the child at home. Selected participants were contacted by the researcher (counselor), and interviewed in a face-to-face to screen for eligibility. Inclusion criteria for caregivers were: currently smoked; lived with the child at least 5 days per week; self-reported as smoking five or more cigarettes a week in front of the child and able to speak Mandarin Chinese or Changsha dialect; having a telephone or mobile phone; and gave signed informed consent or verbal consent for participation. Exclusion criteria for caregivers were: using coal burning for cooking or heating in the home; living away from the child; had lived in the city for less than 6 months; had difficulty in communication or had no phone; and refused participation or child had a serious disease such as heart disease, mental illness and severe asthma. 

**Figure 1:**
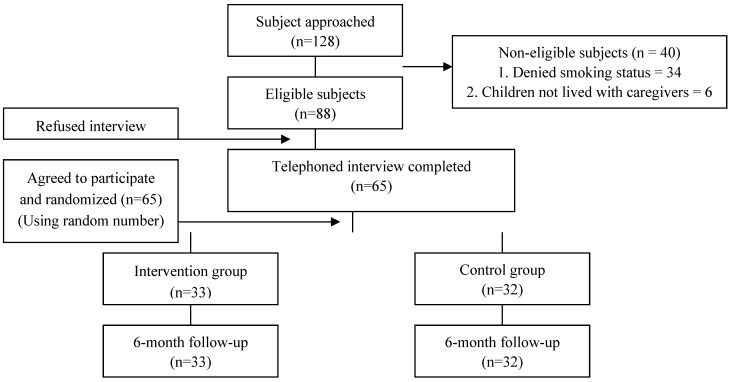
Diagram showing the selection process of participants.

Between December 2012 and January in 2013, 128 families were recruited, of which 88 families were eligible and 23 families refused to participate in the study. After exclusions, a total of 65 smoker caregivers and their children were finally recruited to the study and were randomly assigned in pairs to the intervention (*n* = 33) or control (no intervention) (*n* = 32) group. The sample was composed of 65 participants who were followed-up for 6 months. The follow-up response rate among participants was 100% at 6 months in both groups (Figure 1 shows the process of recruitment participants for the study).

### 2.2. Baseline

At baseline, the counselor conducted an open interview to assess the caregiver’s knowledge of smoking and ETS exposure so as to determine the priorities for behavior change methods. Then we asked caregivers to complete a questionnaire about their child’s exposure to smoke, including frequency, time, and place (e.g., in the home, car, or other places). 

### 2.3. Interventions

#### 2.3.1. Health Education Classes

Health education classes were provided for the kindergarten-age children. All children and their caregivers that were randomly assigned to the intervention group participated in classes, while the families in the control group did not. The classes, entitled “Smoke-free in my childhood” were conducted as part of the activities of an extracurricular club around ETS topics; they were developed specifically for the study and consisted of six sessions over 6 months. The main emphasis was on communicating with caregivers using emotional persuasion strategies, and the secondary aim was to avoiding ETS exposure from strangers. Teachers were trained to coordinate the intervention. Children were encouraged to persuade smoker caregivers to change their behaviors in an effective way by learning special skills. The course included books, role play, manual training, animations, lectures, posters, and presentations by trained teachers. Every class lasted 25 min and two classes were held every month. The home component, which included a bookmark, a card, and a sign, was sent home with the children in individually labeled packages about a month after the preschool intervention was launched. A bookmark, a card and a sign that said no smoking was provided as small reminders for caregivers. The children in the intervention group also brought some materials home about quitting and ETS. Meanwhile, we provided the skills and tips about issues such as managing cravings, keeping busy, finding new things to do, and so on. Although the materials about reducing ETS exposure mainly stressed protecting the child, especially at home, the materials also included strategies to change the smoking behavior of caregivers, such as not smoking in front of their child, smoking outdoors instead, reducing the amount of cigarettes kept at home, and snacking on carrots, pickles, sunflower seeds, apples, raisins or sugar-free gum as an alternative to smoking. Children in the control group did not receive materials about smoking cessation of ETS.

#### 2.3.2. Smoking Cessation Counseling

Treatment consisted of one lecture and five monthly counseling sessions (in-person at the school) over 6 months, together with educational materials and text messages to support caregivers in smoking cessation. Sessions were divided between how to reduce ETS exposure at home and the process of quitting. In the first month, a large-scale lecture was organized to appeal to smoker caregivers and encourage them to pay more attention to protecting their children’s health. Counseling was tailored to the needs of individual clients, which were determined by their smoking status, physical dependence, and the perceived barriers to quitting. The counseling was based on the stages of change component of the Trans-theoretical Model of Behavior Change. The motivational interviews used the FRAME (feedback, responsibility, advice, menu, empathy, and self-efficacy) method to enhance client’s motivation to change, and addressing physiological craving, psychological dependence, and sociocultural factors in relation to tobacco dependency. 

Stage-matched materials were used for smokers as described by Kohrman *et al*., which was published (in Chinese) by the Peking Union Medical College Press in 2007 [23]. These were designed to target smokers at different stages of readiness to stop smoking. A self-help handbook which included three different themes of smoking and ETS exposure was given to caregivers every 2 months and leaflets every month. We also sent motivational text messages by mobile phone and the QQ app (an instant messaging chat tool). Messages were sent 5× daily for 4 weeks and 3× weekly for 16 weeks. The text messages were individually tailored with advice, support and distraction techniques delivered in informal language. The text messages also aimed to remind and support caregivers to set a quit day and take action as quickly as possible. Additionally, we invited the wives of the participants to these activities in order to help remind husbands to stop smoking and increase the influence of the intervention. 

#### 2.3.3. ETS Exposure Counseling 

We used ETS exposure counseling procedures based on the Protective Motivation Theory [24], which has been shown to be effective in previous trials [16,25,26]. This included interviews in which caregiver role models discussed their motivations for protecting children from ETS exposure, the methods which they used to reduce ETS exposure, how ETS exposure prevention was incorporated into their daily routine, and how they overcame any impediments to practicing ETS exposure protection. All counseling sessions, included quitting smoking and reducing ETS exposure, were implemented in parallel during the whole intervention procedure. 

#### 2.3.4. Feedback

We provided information about the child’s urine cotinine concentration as a feedback for caregivers. In this way caregivers would clearly understand the child’s degree of exposure to ETS and be enabled to made decisions about how to avoid it. The counselor contacted caregivers to inspire their motivation to change (e.g., motivation interviews) or give some suggestions of behavior change (e.g., smoke when you are not with your child; smoke with your friend but not at home; remove ashtrays and lighters inside your home; place non-smoking stickers in your home and car; smoke outdoors and away from your child). Finally, we helped caregivers to set short and long-term goals for reducing their child’s ETS exposure (e.g., decreasing the number of smoked cigarettes every day; complete or partial smoking bans in the home and car; quitting smoking).

### 2.4. Control Group

The control group participants underwent all of the study assessments, but they did not receive smoking cessation and ETS exposure counseling. The control group children also did not participate in the health education classes or receive the educational materials or lectures. However, when the 6-month study period finished, the counselor gave interested caregivers from the control group self-help guidance on smoking cessation and a tailored advice letter based on the individual’s situation. The children in the control group also received the same health education classes and education materials after the study period had elapsed. 

### 2.5. Follow-Up

After 6 months, both intervention and control groups was followed up for a final assessment. All participants completed a structured questionnaire and urinary cotinine levels of children at the baseline and after 6 months. All follow-up counseling lasted for an average of 30 min. 

### 2.6. Outcome Measures

#### 2.6.1. Children’s Urine Cotinine Concentrations

In both intervention and control groups, 50 mL urine samples were collected from children, at baseline and 6-month follow-up, into a standard urine collection vial. Caregivers were trained to collected urine samples on Monday morning at home. The first urine of the child was collected in the morning after waking up and stored in the home freezer. When the caregivers sent the child to preschool, they took the urine sample to the kindergarten within 30 min and placed it in a portable icebox. Samples were frozen at −20 °C and packed in dry ice for shipping to the Hunan Academy of Inspection and Quarantine for analysis of cotinine concentration by gas chromatography-triple quadruple mass spectrometry with a limit of quantification (LoQ) of 0.1 ng/mL. The laboratory staff were blinded to the participants’ identity and group assignment. 

#### 2.6.2. Self-Reported ETS Exposure of Children by Caregivers

Exposure was defined as the number of cigarettes smoked while the child was in the same space or 3 m distance from the smoker. The main measures of whether smoking behavior led to ETS exposure of the child (smoked in front of child, smoked with child at home, and smoked around the child at weekdays and weekend) were determined by self-reporting by caregivers. Other measured variables included: total exposure from caregivers and from smokers outside the home during the past 7 days; and the implementation of smoking regulations at home (complete restriction of smoking at home, partial restriction of smoking at home, and no restriction of smoking at home).

#### 2.6.3. Caregivers’ Self-Reported Smoked Status

The smoking status of caregivers, based on self-reporting, were obtained at the baseline and after 6 months. The 7-day point prevalence of smoking cessation was defined as not smoking during the previous 7 days. The 24-hour point prevalence smoking cessation was defined as not smoking during the previous 24 h. To examine the test–retest reliability of our measures, the questionnaires were re-administered to a random sample of 14 caregivers 2 weeks after baseline. Considering that the intervention could affect the test-retest reliability, we implemented the intervention after the test-retest measurements were completed. 

### 2.7. Sample Size and Power

Our sample size calculation was based on a national survey of individuals in China aged 15–69 years in 1996 [27]. We assumed that the quit rates in the current study would be 4% and 29% in the control and intervention group, respectively. This yielded a sample size of 60 (30 in each group), based on a significance level of 5% and power of 80%. Considering a 5% loss rate during the follow-up period, the total sample required was 63 participants.

### 2.8. Statistical Analysis 

The internal reliability was reported using Cronbach’s alpha and the test-retest reliability of caregivers was reported using Pearson’s correlation. Independent *t*-tests and χ^2 ^tests were used to compare the demographic data of the two groups. To compare the number of cigarettes smoked per week between the two groups, we used the Mann-Whitney test and rates of cessation of smoking were compared by Pearson chi-squared test. The children’s urinary cotinine concentrations were compared between the two groups using the Mann-Whitney test. We reported both crude and adjusted (for the variables) odds ratios (ORs) with 95% CI for all the outcome measures. Data were entered into *Epi Info* and analyzed using *SPSS*, version 17.0 (SPSS Inc., Chicago, IL, USA). A *p*-value of <0.05 was accepted as statistically significant.

### 2.9. Group Assignment

The unit of randomization was the individual family. A computer-generated randomization table was used. Randomization information was kept from the study counselor until the baseline assessment was completed.

## 3. Results

### 3.1. Baseline Characteristics 

Of the male caregivers (*n* = 65), most were fathers (92.3%), married (96.9%), with an education level of secondary school or above (95.4%), currently employed (76.9%), a monthly household income of RMB 3000 (US$ 1 = RMB 6.23) or above (75.4%) and daily smokers (100.0%). The mean number of cigarettes smoked per day was 27.2 (median = 25.0). 

**Table 1:** The characteristics of study participants at the baseline.

Characteristics	Intervention Group (*n* = 33) n (%)	Control Group (*n* = 32) n (%)	χ^2^/*t*	*p*
Caregivers’ age (years)	37.61 ± 8.73	35.81 ± 5.97	0.964	0.339
Caregivers			0.295 ^a^	1.000
Father	30 (90.9)	30 (93.8)		
Others (e.g., sibling, grandparent)	3 (9.1)	2 (6.2)		
Caregivers’ ethnicity			0.001^ b^	0.971
Han ethnicity	31 (93.9)	29 (90.6)		
Other	2 (6.1)	3 (9.4)		
Marital status			1.871^ a^	1.000
Married	32 (97.0)	31 (96.9)		
Divorced/separated	1 (3.0)	1 (3.1)		
Occupation status			0.133	0.935
Technical employed	10 (30.3)	11 (34.4)		
Service employed	15 (45.5)	14 (43.8)		
Others	8 (24.2)	7 (21.8)		
Education attainment			2.854^ a^	0.483
Primary or below	3 (9.1)	0 (0)		
Secondary	3 (9.1)	3 (9.3)		
Matriculation	12 (36.4)	14 (43.8)		
Tertiary or above	15 (45.5)	15 (46.9)		
Monthly family income (RMB)			0.889	0.641
≤2999	9 (27.3)	7 (21.9)		
3000–5999	17 (51.5)	15 (46.9)		
≥6000	7 (21.2)	10 (31.2)		
Home size (m^3^; mean ± SD)	91.7 ± 35.0	92.5 ± 30.0	–0.09	0.928
Children’s mean age (years)	6.18 ± 0.46	6.17 ± 0.40	0.614	0.907
Children’s sex			0.675	0.798
Boy	20 (60.6)	21 (65.6)		
Girl	13 (39.4)	11 (34.4)		

**^a^** Fisher’s exact test; **^b^** Continuity correction.

At baseline, no group differences were found for any of the demographic variables shown in Table 1. Children’s baseline urinary cotinine concentrations were not significantly different between two groups (*p* = 0.953; Table 2).

**Table 2:** Children’s environmental tobacco smoke (ETS) exposure from caregivers and other smokers, and urinary cotinine levels, at baseline and post-intervention (after 6 months).

Variable	Baseline	Post-Intervention
Children’s urine cotinine concentration (ng/mL)		
Intervention group	1.72 (1.53–1.92)	1.29 (1.09–1.50)
Control group	1.74 (1.53–1.95)	1.78 (1.55–2.01)
Statistics	Z = −0.059; *p* = 0.953	Z = −3.136; *p* = 0.002
Children’s ETS exposure from caregivers (number of cigarettes/week)		
Intervention group	3.45 (3.04–3.87)	2.05 (1.52–2.59)
Control group	3.54 (3.17–3.90)	3.42 (3.04–3.79)
Statistics	Z = −0.584; *p* = 0.559	Z = −3.752; *p* = 0.001
Children ETS from outdoor smokers number of cigarettes/week)		
Intervention group	0.83 (0.47–1.18)	1.02 (0.86–1.18)
Control group	0.86 (0.63–1.09)	1.20 (1.05–1.35)
Statistics	Z = −1.025; *p* = 0.305	Z = −2.154; *p* = 0.031

Note: Values are geometric means with 95% confidence intervals (95 % CI).

The response rates for the baseline and 6-month follow-up surveys were 100% (65/65), with no significant differences in the response rates between study groups at either time point. Every participant in the intervention group completed five counseling sessions, and every child participated in the health education classes about ETS. In the same way, every participant in the control group completed all measurements and samplings. There were no drop-outs in either groups at both time points.

### 3.2. Reliability and Validity of Caregivers’ Reports 

Internal reliability was assessed as α = 0.946, and correlation coefficients for the caregivers’ reports at the 2-week retest were* r* = 0.811 (all *p*-values < 0.01). The correlation between smoking and urinary cotinine levels was significantly different between the two time points (r_Spearman_ = 0.573, *p* < 0.001). The validity correlation between smoking in the presence of caregivers and children’s urinary cotinine levels was 0.544. 

### 3.3. Intervention Effects

#### 3.3.1. Concentration of Cotinine in Children’s Urine

**Figure 2:**
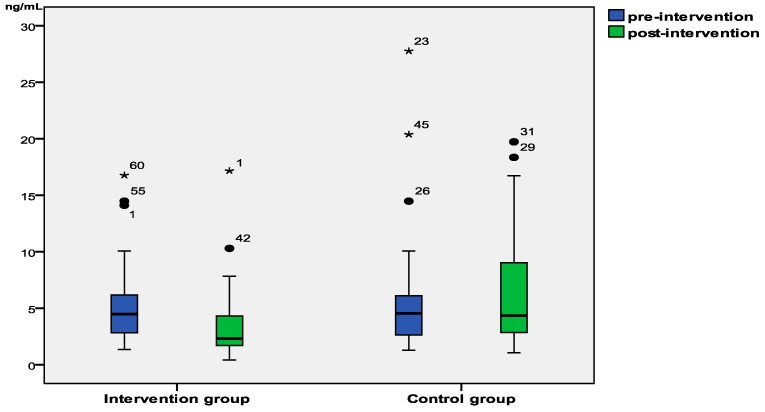
Children’s urinary cotinine levels at the baseline and after the 6-month follow-up period in the intervention and control groups (dot and star represent the outliers).

There were no differences in urinary cotinine levels at baseline between the intervention and control groups (1.72 ng/mL and 1.74 ng/mL respectively; *Z* = −0.059, *p* = 0.953). However, the urinary cotinine level of children in the intervention group was significantly lower than that of the control group children after 6 months (1.29 and 1.78 ng/mL respectively; *Z* = −3.136; *p* = 0.002; Table 2). The cotinine levels had decreased 25.0% in the intervention group and increased 2.2% in the control group (Figure 2).

#### 3.3.2. Smoking in the Presence of Children

Table 2 shows the mean smoking rates of caregivers and others and the children’s ETS exposure at each measure. After 6 months, children’s exposure to smoking by caregivers showed a statistically significant difference between the intervention and control groups (*p* = 0.001). After 6 month, the reported mean ETS exposure from caregivers had decreased 40.6% from baseline among the intervention group and 3.4% among controls. After 6 month, children’s reported exposure from outdoor smokers showed a statistically significant difference between the intervention and the control groups (*p* = 0.031), but after 6 months children’s exposure from outdoor smoke had increased 22.9% among the intervention group and 39.5% among controls. 

#### 3.3.3. Quitting Outcomes and Household Smoking Policies

Table 3 shows that, at the 6-month follow-up, the self-reported 7-day point prevalence quit rate was significantly higher in the intervention group (34.4%, 11/33) than the control group (0%, 0/32) (*p* < 0.001). The crude OR of quitting was 1.12 (95% CI: 1.01–1.23). The OR for 7-day smoking abstinence after adjusting for the variables that differed between the intervention and control group was 1.13 (95% CI: 1.02–1.26). Table 3 also shows that a greater proportion of those in the intervention group had quit smoking (based on self-reported 24 h smoking abstinence) than in control group. There was no statistically significant difference between the intervention and control groups. After adjusting for the variables the OR for self-reported 24-hour smoking abstinence showed no significant difference between the intervention and control groups (0.98; 95% CI: 0.93–1.03). For the other measures, the ORs were as follows: implementation of complete restriction of smoking at home (adjusted OR = 1.1), partial restriction of smoking at home (adjusted OR = 1.0), and no restriction of smoking at home (adjusted OR = 0.9). The number of families in the intervention group that had a partial restriction of smoking at home was higher than that of the control group. 

**Table 3:** Unadjusted and adjusted odds ratios (ORs) for quitting outcomes using different outcome indicators in the intervention and control groups at the 6-month follow-up.

Variable	Intervention (*n* = 33) *n* (%)	Control (*n* = 32) *n* (%)	Unadjusted-OR (95 % CI)	Adjusted-OR (95 % CI) ^a^
Self-reported 7-day quit rates	11 (33.33)	0 (0.00)	1.12 (1.02–1.22)	1.13 (1.02–1.26)
Self-reported 24 h quit rates	32 (96.97)	20 (62.50)	0.96 (0.91–1.01)	0.98 (0.93–1.03)
Complete restriction of smoking at home ^b^	9 (27.27)	4 (12.50)	1.10 (1.01–1.12)	1.15 (1.04-–1.23)
Partial restriction of smoking at home ^c^	21 (63.64)	12 (37.50)	1.04 (0.99–1.10)	1.03 (0.98–1.09)
No restriction of smoking at home ^d^	13 (39.39)	26 (81.30)	0.99 (0.95–1.03)	0.99 (0.96–1.04)

**^a^** Adjusted for variables: age, cigarettes per day smoked by caregiver, number of years smoked and urinary cotinine. OR = odds ratio, CI = confidence interval; **^b^** No smoking at all inside the home; **^c^** No smoking within 3 m of the child at home; **^d^** No smoking restrictions inside the home.

#### 3.3.4. Measurement of Smokers’ Behavior Change and Children’s Urinary Cotinine

The data on the other measures—decrease in cigarettes smoked by caregivers in the presence of children, rates of quitting smoking, and children’s urinary cotinine levels—after the intervention are presented in Table 4. There were statistically significant differences in the number of cigarettes smoked by caregivers in the presence of children and in self-reported 7-day quit rates. There was a great reduction achieved in self-reported 7-day quit rates (*p* = 0.003); the average number of cigarettes smoked by caregivers in the presence of children also decreased significantly (*p* = 0.005). 

**Table 4:** Caregiver’s smoking behavior changes and children’s urine cotinine levels.

Variables	*n* (%)	Mean ± SD	*t*	*p*
Decrease of cigarettes smoked by caregivers in presence of children (number of cigarettes/week) ^a^				
≤1.42	34 (52.3)	0.01 ± 0.58	−2.903	0.005
>1.42	31 (47.7)	0.40 ± 0.48		
Self-reported 7-day quit rates				
Yes	11 (16.9)	0.65 ± 0.61	3.081	0.003
No	54 (83.1)	0.11 ± 0.51		
Self-reported 24-h quit rates				
Yes	13 (20.0)	0.45 ± 0.46	−1.089	0.280
No	52 (80.0)	0.24 ± 0.59		

**^a^** Geometric mean.

## 4. Discussion

The proposed health education intervention was intended to raise children’s awareness about measures they can take to protect themselves and to act as strategy to avoid ETS exposure, while at the same time alerting caregivers and educators to the importance of protecting children’s health. We compared a group in which both the caregivers and the children were counseled/educated and a group in which neither caregivers nor children received the intervention. Clearly, there are other combinations and permutations of interventions, which the current study design has not tested. The following discussion therefore focuses on what can be learned from the two specific groups that we chose to compare.

### 4.1. Urinary Cotinine Levels of Children

Cotinine is a major metabolite of nicotine and is considered the best measure of nicotine consumption and exposure [28,29]. Our study developed a novel intervention using personalized biofeedback data (children’s urinary cotinine levels) and children as health agents aimed at encouraging caregivers to reduce children’s exposure to ETS in their home, and explored its feasibility, utility, and acceptability. To the best of our knowledge, this is first study to report urinary cotinine levels in children aged 5–6 years exposed to ETS and of preschool children participating in ETS-related classes in China. The urinary cotinine levels of children in the intervention group were significantly lower than that of the control group children after 6 months. These results are in agreement with previous studies [10,30]. Compared with other studies, the urinary cotinine levels in children exposed to passive smoking in China are lower than children in other countries [14,31]. However, it was noted in 2004 that about 60.2% of men in China were smokers and the children were the major victims of passive smoking, especially at home [32]. One of the most effective means of lowering the impact of ETS on children is parental smoking cessation, especially by the mother [33]. However, most women in China, especially those with children, are not smokers [12,32,33,34,35]. Therefore, children are mainly exposed to ETS by male smokers in China, especially their fathers. 

### 4.2. Smoking Cessation

Smoking cessation by parents and other caregivers is the best way to protect children from ETS exposure at home [36]. The self-reported 7-day quit rate in the intervention group in our study was higher than the reported quit rates in a randomized controlled trial (Hong Kong) [37], but lower than Krieger *et al*.’s study also in the US [38]. A recent systematic review showed that multiple contacts timed around a quit attempt were effective in encouraging smoking cessation [39]. Our results indicated that a combination of interventions for caregivers along with counseling and classroom-based health education for their children may be useful to help male caregivers to change their household smoking behaviors. Previous studies have shown that children are not merely recipients of health education, but can also contribute to disease control and prevention strategies by playing the role of health change agents in both the community and home [16,18]. Our findings suggested that engaging children as health agents had a substantial impact not only on children, but also on their smoking family member in improving their knowledge about ETS exposure and changing smoking behaviors. Interviews with caregivers in the current study showed that the children reinforced the fact that they should not smoke and take measures to avoid smoking as indicated by the educational material provided. This could even lead to smoking cessation. None of the preschoolers dropped out of the trial, nor did any of the caregivers. The findings suggest that health education in kindergartens regarding the harm of ETS exposure may prompt children to remind and persuade caregivers not to smoke at home, and also that communication between caregivers and teachers is a way to protect children from the harms of ETS exposure at home. 

### 4.3. Smoking Restrictions

In this study we reported several other outcome measures, some of which improved for participants in the intervention group. Behavior change for smokers can be assessed not only from their success in quitting smoking but also from other measures, such as reducing smoking in the home, implementing partial/complete restriction of smoking in the home, or by making other positive changes; these could also be informative. However, the complete restriction of smoking at home is difficult to achieve. In China, hosts show hospitality through the tradition of providing cigarettes, drinks and tea to guests. These results are supported by other studies [40,41]. Another factor to consider is third-hand smoke, which refers to the components of ETS that stick to indoor surfaces and persist in the environment [1,42,43,44]. Third-hand smoke may act as another source of information about possible toxic substances in the home for families with young children and may motivate caregivers to change their smoking behavior to better protect the health of their children. More importantly, research has found that THS exposure to a toddler occurs by them mouthing fabrics used in toys, drapes and upholstery that are not frequently washed; these fabrics can accumulate smoking-related toxins over a long period of time and the resulting concentrations can be sufficiently high to cause harm [44]. It is well-known that there is no risk-free level of exposure in environmental tobacco smoke. The new information and research evidence showed that it is very important and informative to guide and implement further intervention in the future study.

### 4.4. Strengths and Limitations

This study had several major limitations. First, more combinations of interventions exist which the current design did not consider. It was not possible to distinguish whether the main contribution to the success of the intervention was due to the caregivers, the children or both. Secondly, the intervention was given at a time when caregivers may have been concerned about the effects of smoking on their child’s health and thus have been primed to accept the counselor’s advice on quitting. Therefore, these results might not be generalizable to other populations where such a factor does not apply. Thirdly, the post-intervention research period lasted only for 6 months and it is not certain what proportion of those who quitted at 6 months would continue for a longer duration. On the other hand, a strength of the study was that there were no dropouts among the participants and all of them completed the intervention process. Fourthly, researchers have established the newly defined issue of third-hand smoke [44], but data about this was not collected in this study. Future studies will quantify ETS exposure as measured by airborne PM_2.5_ at different time points and locations within the home. Fifthly, because the family was the unit of randomization, there was a risk of contamination from teachers discussing smoking exposure to a child in the control group. However, as a preliminary explorative trial for the use of ETS-related interventions in preschool children, the effects of intervention were inspiring.

## 5. Conclusions

The improved knowledge about smoking from the intervention and the rate of quitting smoking practice could be linked to a decrease in the prevalence of exposure of preschool children to ETS at home. This study is one of the first to focus on male smokers, especially fathers. These results suggest that more children and families could benefit if such an intervention were implemented in parallel in the preschool setting and at home. 
